# Adverse Effects of Immunoglobulin Therapy

**DOI:** 10.3389/fimmu.2018.01299

**Published:** 2018-06-08

**Authors:** Yi Guo, Xin Tian, Xuefeng Wang, Zheng Xiao

**Affiliations:** ^1^Department of Neurology, The First Affiliated Hospital of Chongqing Medical University, Chongqing Key Laboratory of Neurology, Chongqing, China; ^2^Center of Epilepsy, Beijing Institute for Brain Disorders, Beijing, China

**Keywords:** immunoglobulin, adverse effects, risk factors, preventive measures, premedication

## Abstract

Immunoglobulin has been widely used in a variety of diseases, including primary and secondary immunodeficiency diseases, neuromuscular diseases, and Kawasaki disease. Although a large number of clinical trials have demonstrated that immunoglobulin is effective and well tolerated, various adverse effects have been reported. The majority of these events, such as flushing, headache, malaise, fever, chills, fatigue and lethargy, are transient and mild. However, some rare side effects, including renal impairment, thrombosis, arrhythmia, aseptic meningitis, hemolytic anemia, and transfusion-related acute lung injury (TRALI), are serious. These adverse effects are associated with specific immunoglobulin preparations and individual differences. Performing an early assessment of risk factors, infusing at a slow rate, premedicating, and switching from intravenous immunoglobulin (IVIG) to subcutaneous immunoglobulin (SCIG) can minimize these adverse effects. Adverse effects are rarely disabling or fatal, treatment mainly involves supportive measures, and the majority of affected patients have a good prognosis.

## Introduction

**Figure 1:**
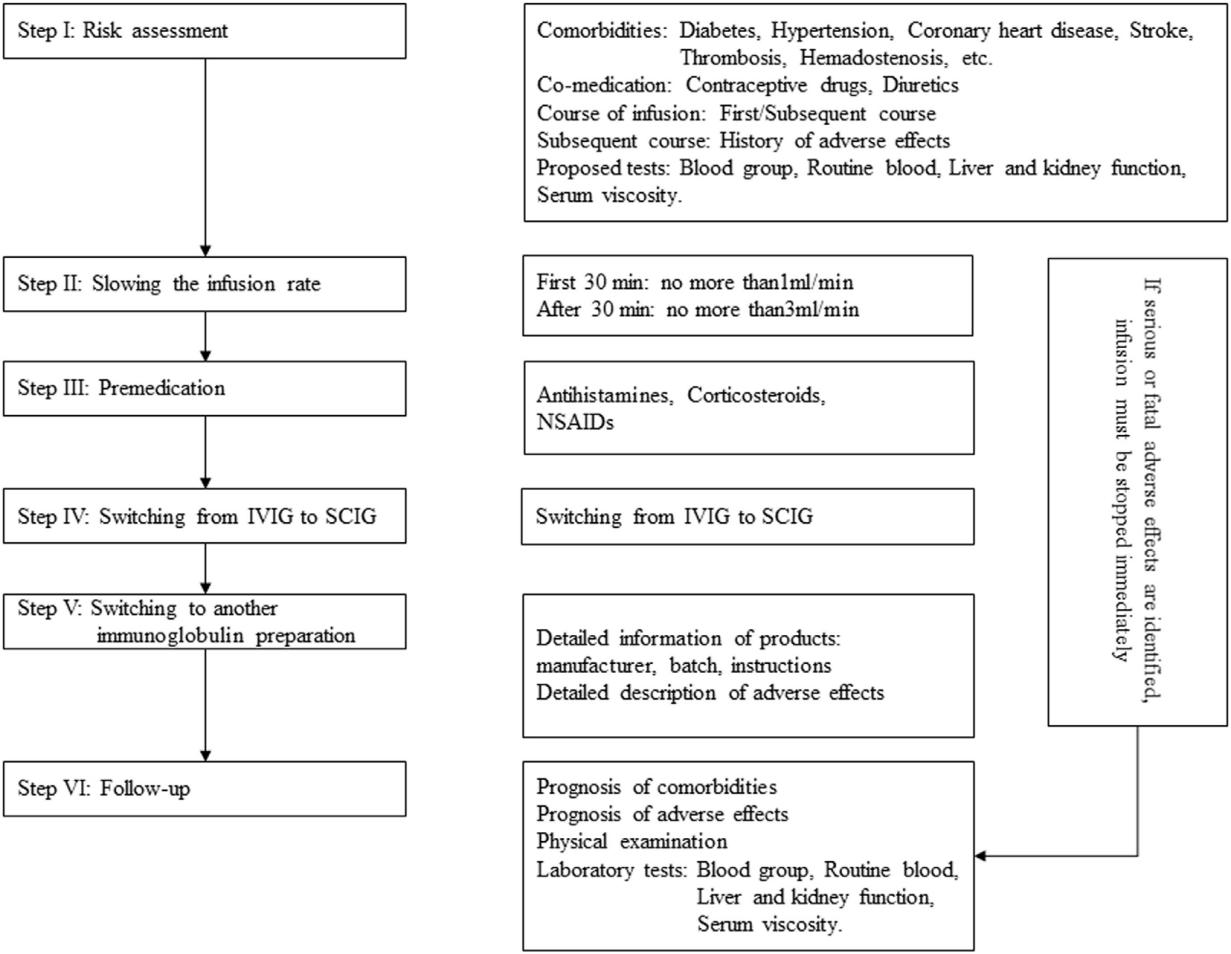
Process that minimizes or prevents immunoglobulin-associated adverse reactions.

Immunoglobulin, also known as gamma globulin, is a therapeutic preparation comprising pooled blood donated from large numbers of healthy people. IgG is the main component of immunoglobulin, but it also contains small amounts of IgA and varying trace amounts of auxiliary materials (maltose, sucrose, etc.) (1). Applications involving immunoglobulin have expanded to include treatment for immunodeficiency diseases, idiopathic thrombocytopenic purpura (ITP), Kawasaki disease, and neurologic disorders (including Guillain–Barre syndrome, chronic inflammatory demyelinating polyneuropathy, myasthenia gravis, multiple myositis, multiple sclerosis, and autoimmune encephalitis) (2–8). Although immunoglobulin is well tolerated, adverse effects do occur. The majority of these adverse effects are mild and alleviated after infusion withdrawal, but some rare side effects are serious, including aseptic meningitis, renal impairment, thrombosis, and hemolytic anemia (9). In this paper, we reviewed the incidence, risk factors, clinical manifestations of and preventive measures for adverse effects related to immunoglobulin. The processes employed to minimize adverse reactions are briefly addressed in Figure 1.

## Historical Perspective

In 1890, the German scholar Behring won the 1901 Nobel Prize in medicine for developing a serum therapy for diphtheria, representing a new chapter in the search for immunotherapies (10). In 1941, Cohn et al. (11) successfully developed a process for the large-scale production of human immunoglobulin. Next, immunoglobulin was widely used during World War II. In 1952, Bruton (12) was the first to use immunoglobulin to treat a patient identified as immunodeficient, and it later became a standard therapy for immunodeficiency diseases. Intramuscular immunoglobulin preparations were not widely applied because of their poor tolerance. Hence, many scholars began to explore intravenous immunoglobulin (IVIG) preparations. Until 1979, IVIG was approved to treat immunodeficiency disease by the American Food and Drug Administration (13). Imbach et al. (14) then introduced IVIG as a treatment for ITP, with desirable effects. Since then, IVIG has been widely used for an increasing number of diseases.

## Incidence of Adverse Effects

Patients who receive immunoglobulin therapy are often treated with immunoglobulin in repeated infusions over a long period of time, and the incidence of adverse effects related to immunoglobulin varies across a wide range. For example, in the study by Matsumoto et al. (15), 14 of 567 (2.5%) patients experienced adverse effects during infusion with IVIG. However, another study reported that 87.5% (14/16) of patients had adverse effects during treatment with repeated infusions of IVIG (16). The majority of studies have focused on the rate of adverse effects in patients receiving multiple infusions over time; however, information regarding the rate of a single infusion is scarce. For subcutaneous immunoglobulin (SCIG) preparations, many studies suggested that the adverse effects of SCIG were much lower than those of IVIG, and the incidence varied across a wide range. However, these studies cannot reveal the occurrence of adverse effects due to multiple interfering factors, such as differences in immunoglobulin preparations, individual differences, or study design variations.

Variations in immunoglobulin brands used may be the main cause for this lack of information regarding the occurrence of adverse effects, considering that different immunoglobulin formulations can have different adverse event profiles. Many clinical trials aimed to evaluate the safety of investigational immunoglobulin products that were not standardized with respect to data collection and provide a definition for adverse effect. Different studies focused on various segments of the population, and these patients had many diseases and fluctuating risk factors. Furthermore, patients who tolerated the infusions may have been shifted from the hospital to home-based infusion therapy, thereby explaining the broad range of adverse effects.

Study design variations also affected the rate of adverse effects. Most trials have involved a limited sample size, and few trials that were performed to support licensed marketed products included a control group. The lack of a control group increases the difficulty involved in unambiguously ascribing causality, and some studies did not report the frequency. In the study design, we advised investigators to predefine the time frame over which adverse effects are considered temporally associated with the infusion of the product (i.e., within 24, 48, or 72 h of the end of the infusion) in the protocol. Based on prior experience with the same products and to include all the adverse effects, *a priori* algorithm for assigning causality was developed to observe adverse effects and preidentify a list of these adverse events, which are presumed to be related to the administration of the test products.

Analyzing the epidemiology of adverse effects in clinical research is important. For patients receiving long-term IVIG replacement, the rate of adverse effects should be calculated according to the infusion times (per infusion, not per patient). Furthermore, a nationwide database for immunoglobulin-related adverse effects should be created, but the following factors that affected the passive reporting of post-marketing surveillance data in this database should be taken into consideration: (1) Some adverse effects occurring during the IVIG infusion may not be associated with IVIG; (2) some adverse events can be duplicated and result in double counting, especially adverse events that last over an extended period of time; and (3) substantial underreporting is likely to occur as it is a voluntary system.

## General Risk Factors

### Immunoglobulin Preparation-Related Risk Factors

A high concentration of IgA and anti-Rh blood group, D antigen (RhD) increases the occurrence of immunoglobulin-related adverse effects. Manlhiot et al. (17) found that adverse effects were reported more often in patients treated with immunoglobulin products that contained a concentration of IgA higher than 15 µg/ml (15 VS 8%). Similarly, a high titer of anti-RhD also increased the occurrence of adverse effects; therefore, the level of anti-RhD should be maintained as low as possible (18, 19). However, preparations produced by different manufacturers have different excipients that may increase the rates of specific adverse reactions (20) (Table 1).

**Table 1:** Components of immunoglobulin products associated with adverse effects.

Component	Patients with increased risk
Sucrose	Patients with renal failure
Glucose	Patients with diabetes
Maltose	Patients with glucose fluctuation
Sorbitol	Patients with hereditary fructose intolerance
High IgA	Patients with risk of anaphylaxis

### Patient-Related Risk Factors

Patients who developed adverse effects during a previous course and those receiving a first infusion are at an increased risk of adverse effects. Sherer et al. (21) found that 9 of 10 (90%) patients who experienced an adverse effect during the first treatment course also had adverse effects during subsequent courses. A survey conducted in Iran verified that the risk was higher in patients receiving a first course than in those receiving subsequent treatment courses (16.2 VS 6.9%, respectively) (22).

Some studies have suggested that IgA-deficient patients may be at a higher risk of adverse effects. Iranian researchers found that the incidence of immunoglobulin-induced adverse effects was higher in patients with primary antibody defects, especially those with low levels of IgA (22, 23). In contrast, Rachid and colleagues found that the role of IgA-deficiency in anaphylaxis in patients during immunoglobulin therapy remains controversial (24, 25). Therefore, immunoglobulin infusion should never be withheld from IgA-deficient patients.

## Classification of Adverse Effects

Adverse effects are classified as immediate or delayed depending on the time of occurrence. Immediate adverse effects mainly include flu-like syndrome, dermatologic side effects, arrhythmia, hypotension, and transfusion-related acute lung injury (TRALI). Immediate side effects are categorized as mild, moderate, and severe. Mild adverse effects include light headache, fever, chills, and fatigue; they are alleviated when the infusion is slowed down or when antihistamines and nonsteroidal anti-inflammatory drugs (NSAIDs) are administered. Moderate adverse effects include chest pain, anhelation, vomiting, arthralgia, and severe headache; these effects require the infusion to be discontinued or antihistamines and NSAIDs to be administered. Severe adverse effects include hypertension, anaphylaxis, bronchospasm, and altered consciousness; these adverse effects require the infusion to be stopped immediately and for corresponding medical attention to be provided (22).

### Immediate Adverse Effects

#### Flu-Like Symptoms

Flu-like symptoms are the most frequent adverse effects. These include flushing, nausea, fatigue, fever, chills, malaise, and lethargy. One retrospective study showed that 14 of 16 (87.5%) patients developed flu-like symptoms during immunoglobulin administration (16). Bichuetti-Silva et al. (26) found that flu-like symptoms account for more than 80% of immunoglobulin-induced adverse effects. These symptoms always occur within the first hour of infusion, and some adverse effects (such as fever or fatigue) may also arise within 24 h. The mechanism underlying these symptoms remains unclear, but it may be associated with the presence of cytokines, such as IL-6, TNF-α, prekallikrein activator, and kallikrein, in immunoglobulin products. The solution media and complement activation of an immunoglobulin preparation may also represent causes of these effects (27, 28). The majority of these symptoms are associated with rapid infusion and develop during the initial period of infusion. Hence, it is recommended that infusion should start at a slow rate for the first 30 min (29).

#### Dermatological Adverse Effects

The incidence of immunoglobulin-related dermatological adverse effects is nearly 6% (30, 31). The manifestations of dermatological adverse effects vary among individuals and can include urticaria, spot papules, eczema, pompholyx, lichenoid dermatitis, and desquamation. Epidermolysis is observed in some severe cases, and all of these skin lesions can occur in all parts of the body, although the hands and feet are the most common sites. Gerstenblith et al. (32) found that 62.5% of patients had pompholyx alone or a combination including pompholyx on the hands or feet. The mechanism underlying these dermatological adverse effects is unclear. Most of these reactions develop within 2 weeks of immunoglobulin administration. Interestingly, a significant number of patients with skin lesions had neurological disorders, and the repeated administration of a high-dose immunoglobulin infusion within a short period of time may be the cause of this high preponderance (32, 33). Dermatological adverse effects can be successfully treated with corticosteroids. Some severe cases require hospitalization for further management, but there are no reports of deaths resulting from severe adverse reactions of the skin in immunoglobulin-treated patients. Switching to another batch of immunoglobulin product may also reduce adverse effects to some extent (33).

#### Arrhythmia and Hypotension

Arrhythmia occurring during or after immunoglobulin infusion has been reported in several studies and can include supraventricular tachycardia and bradycardia, while most of the cases had a history of heart disease. In 1997, Savasan et al. (34) showed that arrhythmia developed during IVIG in two children with thrombocytopenia who both had a history of arrhythmia, and the condition was resolved in both cases with antiarrhythmic therapy. In 2015, Tufekci et al. (35) reported that supraventricular tachycardia occurred during IVIG administration in two newborn infants with immune hemolysis. Raheja et al. (36) described a case of asymptomatic bradycardia that occurred after IVIG administration in a female with ITP who reached a lowest heart rate of 30 bpm before returning to baseline levels without any therapy. Although it is not fully understood whether arrhythmia is directly related to immunoglobulin infusion, cardiac monitoring during IVIG infusion is recommended in patients with a history of cardiac disorders.

Hypotension is a rare symptom related to immunoglobulin. Some patients with hypotension also experience anaphylactic shock. Dashti-Khavidaki et al. (22) reported 216 patients who developed adverse effects, and only one of these patients developed hypotension combined with allergy and bronchospasm. Charhon et al. (37) later described a case with hypotension (a decrease in systolic blood pressure from 130–60 mmHg) and an altered mental state during immunoglobulin therapy.

#### Transfusion-Related Acute Lung Injury

Transfusion-related acute lung injury, a serious blood transfusion-related adverse effect with high mortality, manifests with acute respiratory distress and noncardiac pulmonary edema within 6 h of transfusion and is the main cause of blood transfusion-related death. Immunoglobulins are blood products and may also be associated with TRALI. In 2001, Risk (38) first reported a 23-year-old male with multifocal motor neuropathy who developed TRALI following IVIG therapy; the patient’s condition resolved in 5 days with only nasal oxygen and bed rest. In 2008, Ahituv et al. (39) presented an adolescent patient with ITP who developed TRALI during immunoglobulin infusion. In several cases, patients with Sjögren’s syndrome, Guillain–Barre syndrome, lung transplantation, immunodeficiency, or myasthenia gravis have reportedly developed TRALI after immunoglobulin infusion (40–44). Akin to blood-infusion TRALI, immune-mediated processes and the neutrophil-priming hypothesis have been proposed as possible mechanisms (45). TRALI following IVIG is a serious complication that requires urgent treatment. Diagnosing TRALI depends mainly on clinical symptoms that present after blood products are infused in the absence of other evident causes of respiratory insufficiency; a chest radiogram showing diffuse bilateral pulmonary edema is also needed (46). Patients with TRALI often require adjuvant ventilatory therapy and will recover with proper ventilation.

### Delayed Adverse Effects

Delayed adverse effects can be severe or even lethal and affect less than 1% of patients. These events include thrombotic events, neurological disorders, renal impairment, hematologic disorders, electrolyte disturbance, and transfusion-related infection.

#### Thrombotic Events

Thrombotic events are serious adverse effects of immunoglobulin treatment with an estimated incidence of 1–16.9% (47). Daniel et al. (48) reviewed thrombotic adverse events recorded in a large administrative database from 2008 to 2010 and found that 1% (122/11785) of the patients developed immunoglobulin-induced thrombotic events. Ramírez et al. (49) found that thrombotic events affected up to 16.9% of 303 patients who received immunoglobulin infusion. The manifestations of thrombosis, which can occur in arteries, veins, and intracranial vein sinuses, are varied, and arterial thrombotic events (such as stroke, myocardial infarction, and pulmonary embolism) are the most common. A recent review identified 100 cases of thrombotic events related to the administration of immunoglobulin that occurred from 2006 to 2011; among this cohort, 80% of the thrombotic events were stroke and myocardial infarction that occurred within 24 h of completing immunoglobulin administration (50). Risk factors for thrombosis include a first infusion consisting of a large dose, oral contraceptive use, advanced age, prior/current thrombosis, preexisting atherosclerotic disease, elevated serum viscosity, a hereditary hypercoagulable state or ITP. Rajabally et al. (51) found that patients with coronary disease and prior thrombosis who were administered a daily dose ≥35 g of IVIG had a higher risk of thrombotic events. Daniel et al. (48) also found that advanced age (>45years old), prior thrombotic events, and a hypercoagulable state were risk factors for the development of thrombotic events. Moreover, an increasing number of studies have confirmed that patients with ITP are more likely to develop thrombosis when receiving IVIG (52–55). The presence of four or more risk factors seems to be significantly associated with the onset of immunoglobulin-related thrombotic events (51, 56).

Mechanisms that could potentially trigger thrombotic events include an increase in plasma viscosity, the activation of procoagulant factors, vasospasm, autoimmune vasculitis, and an increased platelet count. Increased plasma viscosity contributes most to the occurrence of thrombotic events. Bentley et al. (57) showed that IVIG can cause plasma viscosity to acutely and cumulatively rise across the complete treatment course. Similarly, Baba et al. (58) also found that increased plasma viscosity was associated with IgG concentrations during or after immunoglobulin infusion. In addition, the average IgG half-life among individuals varied from 23 to 30 days and could be even longer in some cases (59, 60). Thrombotic complications can be prevented or minimized by early assessment in patients suspected of being at high risk, and anti-thrombus treatment is needed in patients with thrombotic complications.

#### Neurological Disorders

Neurological disorders associated with immunoglobulin treatment include headache, aseptic meningitis, posterior reversible encephalopathy syndrome (PRES), seizure, and abducens nerve palsy.

Headache post IVIG is a common adverse effect. More than half of patients develop headaches after immunoglobulin administration. Many studies have reported headache as an immunoglobulin-related adverse effect, while no studies have described the characteristics of immunoglobulin-related headache in detail. Headache also has a delayed onset of 6–12 h after an infusion and can last between 24 and 72 h. High-dose immunoglobulin infusion is the main risk factor for headache. Some studies have found that patients with a history of migraine are prone to developing headaches after IVIG infusion (61, 62). Among these studies, the overall incidence of IVIG-related headache in patients with a history of migraine was small, which may be due to the small sample size and patient selection bias. Prophylactic treatment used in several studies included acetaminophen, aspirin, opioids, NSAIDs, propranolol, sumatriptan, and corticosteroids alone or in combination, and all the protocols seemed to be fairly effective in the recruited individuals; however, the best drug for the treatment of immunoglobulin-related headache remains unknown (63). Alternatively, non-pharmacotherapy-related approaches, including a reduction in the infusion rate and switching to an alternative brand of IVIG or SCIG, can also reduce headache to some extent. When a headache lasts for a long time or is resistant to drug therapy, the possibility of aseptic meningitis should not be ignored.

Aseptic meningitis has been identified as an adverse effect of IVIG and affects 0.6–1% of patients. Kemmotsu et al. (64) retrospectively examined 384 patients with Kawasaki disease who received immunoglobulin infusion during 2000–2009 and identified four patients who developed aseptic meningitis, suggesting an overall incidence of 1%. In another retrospectively study, Bharath et al. (65) found that 0.6% (8/1324) of patients developed immunoglobulin-related aseptic meningitis. Several studies have reported that aseptic meningitis appears within 48 h of the initiation of IVIG therapy (64, 66, 67). The most common presenting symptoms of this condition are persistent headache, nausea, vomiting, photophobia, fever, chills, and positive Kernig’s and Brudzinski’s signs. In addition, in affected patients, lumbar puncture typically produces clear cerebral spinal fluid with an increased level of nucleated cells, high protein content and negative culture results (64, 66). Contributing factors include a large cumulative IVIG dose and a history of migraines. Previous studies have suggested that most patients who develop aseptic meningitis receive 1–2 g/kg immunoglobulin therapy (64, 65). Sekul et al. (66) found that patients with a history of migraines are particularly susceptible to developing aseptic meningitis.

Posterior reversible encephalopathy syndrome is another neurological disorder that can develop following immunoglobulin therapy. In 2005, Nakajia (68) reported a patient with Miller–Fisher syndrome who developed PRES following IVIG therapy. Stetefeld et al. (69) and Ribeiro et al. (70) later reported additional patients with Miller–Fisher syndrome who developed PRES during immunoglobulin infusion. The clinical manifestations in these patients include an acute onset characterized by headache, generalized seizure, visual impairment, and an altered mental state (69–71). In all of these cases, complete resolution was achieved after immunoglobulin administration ceased. While PRES is a rare complication of IVIG treatment, it should be considered in all cases with disease-typical MRI findings and clinical manifestations.

Few case reports have described patients with IVIG-associated seizures. In 2003, Kao et al. (72) reported a 37-year-old male with myelopathy who developed repetitive generalized tonic-clonic seizures following IVIG therapy. Later, aseptic meningitis was considered the cause of the seizures, and the patient’s condition was controlled with valproate. In 2014, Bichuetti-Silva et al. (26) identified a separate case of seizure in a patient with common variable immunodeficiency disease following IVIG infusion, and a history of herpetic encephalitis was later recorded.

Immunoglobulin infusion may rarely be the underlying etiology of abducens nerve palsy. Wright et al. (73) reported a patient with renal transplantation who developed abducens nerve palsy during high-dose IVIG infusion. The patient recovered completely after 2 weeks, and aseptic meningitis was considered the underlying cause. Furthermore, two case reports described patients with Kawasaki disease who developed abducens nerve palsy after IVIG therapy (74, 75). However, whether abducens nerve palsy was directly related to immunoglobulin therapy in these cases is unclear.

#### Renal Impairment

Renal impairment following immunoglobulin treatment is a rare but dangerous adverse effect. The incidence of immunoglobulin-associated renal impairment has not been accurately determined. The FDA received information related to 114 cases of immunoglobulin-associated renal impairment or acute renal failure that occurred between 1981 and 1998 (76). Moreover, from 1999 to 2005, the French National Security Agency of Medicines and Health Products recorded 91 cases of renal impairment associated with immunoglobulin infusion (77). The precise mechanism underlying IVIG-related renal impairment remains unclear. Potential mechanisms include the precipitation of immune complexes in the glomeruli, osmotic nephritis, immunological hemolysis-associated acute tubular obstruction, and transient vascular ischemia due to a reduction in renal perfusion (78–80).

Patients with advanced age, diabetes, preexisting renal dysfunction, and dehydration have an increased risk of developing renal impairment following immunoglobulin administration. Renal impairment typically develops within 10 days after the start of immunoglobulin infusion. Oliguria, hematuria, a decreased glomerular filtration rate, and elevated serum creatinine levels are typical manifestations of renal impairment. Serum creatinine levels usually peak around day 5, and oliguric renal failure is more common than other types of renal dysfunction (9, 81, 82). Renal function usually returns to normal after IVIG infusion is discontinued or short-term hemodialysis is performed, but a few patients with immunoglobulin-related renal impairment have developed chronic renal insufficiency or died (83, 84). Previous studies have shown that death occurs in 8–15% of these patients, but the majority of patients who die have severe underlying conditions, such as advanced age, uncontrolled diabetes, or prior renal dysfunction; therefore, the cause of this effect needs further study (83, 85, 86). In patients with renal insufficiency, renal function should be closely monitored both before and after treatment (including serum creatinine, blood urea nitrogen levels, and glomerular filtration rate). If renal function progressively declines, the rate of infusion should be reduced or treatment should be discontinued. Epstein et al. (76) verified that sucrose-containing preparations should be avoided because they are associated with a risk of osmotic nephritis. However, Kim et al. (87) found that sucrose-free IVIG could also result in renal impairment.

#### Hematologic Disorders: Hemolysis and Neutropenia

Hemolysis is an adverse effect related to IVIG administration that occurs in approximately 1.6% of patients but is usually neither recognized nor treated because it lacks clinical symptoms. Hemolysis can result in acute renal failure and thrombosis. In 2008, a case series conducted at Ottawa Hospital identified 16 cases of hemolysis among approximately 1,000 patients who received IVIG infusion (resulting in an incidence of 1.6%) (88). Most cases with hemolysis present no obvious clinical symptoms and are diagnosed with low hemoglobin levels on a blood examination. IVIG infusion-associated hemolysis was observed from 12 h to 10 days after the first infusion of IVIG, with the lowest hemoglobin level occurring between 1 day and 2 weeks after the last IVIG infusion. Hemolysis is a common complication of high-dose IVIG derived from non-group O blood. In a systematic review conducted by Desborough and colleagues, 62 cases of hemolysis were identified, and 97% of those patients had received a high dose of IVIG (at least 2 g/kg). Of those 62 cases, IVIG-induced hemolysis was most common in patients with type A (65%) or AB (26%) blood (89). Several more recent studies have also verified that administration of a high dose of IVIG is a contributing factor in hemolysis (90–92). This effect may be associated with the presence of A and B isoagglutinin (anti-A and anti-B antibodies) in the IVIG product. A recent cohort study found that the risk of hemolysis was lower when donors with high plasma titers of anti-A antibodies were excluded, especially in patients requiring ≥1.75 g IVIG/kg (93). Abnormal laboratory tests that may indicate hemolysis include decreased hemoglobin and haptoglobin levels, increased lactate dehydrogenase levels, and increased hemobilirubin and reticulocyte counts (94). The management plans generally proposed in affected patients aim to slow down the rate of infusion, switch to another IVIG product, or check the blood type for potential indications for hemolysis. Hemolysis is self-limiting in the majority of mild and moderate cases. However, proper blood transfusion is needed in severe cases when a Coombs test or a direct or indirect antiglobulin test is negative (89, 95, 96).

Immunoglobulin therapy can cause hemolysis, but it can also cause neutropenia (97). In 1998, Majer et al. (98) first described neutropenia as a complication of IVIG therapy in children with ITP. Veys et al. (99) also reported two cases of neutropenia following IVIG infusion for ITP. In addition, Matsuda et al. (100) and Bajaj et al. (101) reported that patients with neurological disorders could develop neutropenia with IVIG administration. Currently, immunoglobulin-induced neutrophils have been reported only in case reports. This condition usually occurs within 4 days after infusion and recovers spontaneously without infection in 2 weeks. However, premedication with corticosteroids may be an effective measure to prevent neutropenia (98–103).

#### Electrolyte Disturbance

Electrolyte disturbance is a rare adverse effect of immunoglobulin administration. In 1997, a study that examined variations in serum chemistry among 46 patients receiving IVIG infusion found that sodium and magnesium levels were significantly lower in infused patients (4 and 7% below baseline, respectively) (104). Daphnis et al. (105) retrospectively evaluated a cohort of 66 patients with ITP who received repeated IVIG infusions and found that serum sodium levels fell by 2.7 mmol/l in patients with normal renal dysfunction and 5.7 mmol/l in patients with acute renal failure. These electrolyte disturbances usually have no clinical symptoms, and affected patients generally recover without electrolyte supplementation. With regards to patients with severely compromised renal function, it is recommended that electrolyte levels can be monitored to identify hyponatremia and hyperkalemia.

#### Infection Risk

The long-term safety of immunoglobulin preparations is excellent. Until recently, the majority of physicians believed that IVIG infusion was associated with no risk of infection. Since immunoglobulins are blood products, there will always be a risk of underlying infection, which may be fatal. Until recently, the most commonly reported infection was the hepatitis C virus. In 1994, the FDA and Centers for Disease Control and Prevention received reports of over 100 cases of acute hepatitis virus infection in recipients of IVIG from several countries (Norway, United States, Europe, and Puerto Rico). The brand names Gammagard and Polygam accounted for the majority of the cases of hepatitis C in both the United States and Europe (106, 107). Razvi et al. (108) reported the outcomes of 58 cases with IVIG-transmitted hepatitis C, and the prognosis of these subjects was poor. Since then, no cases of immunoglobulin-induced hepatitis C have been reported.

Apart from immunoglobulin-transmitted hepatitis C, no cases of IVIG-related hepatitis B virus (HBV) have been reported. However, IVIG-related passive transfer of hepatitis B antibodies has also been reported. Several scholars have reported cases in which the patient developed positive HBV core antibodies and surface antibodies after IVIG infusion, and subsequent multiple tests revealed false-positive results due to immunoglobulin (109, 110). Ramsay and colleagues conducted a cross-sectional study that suggested that HBV antibodies (HBsAb and HBcAb) are common in patients receiving IVIG and who have confounding diagnostic results (111). Thus, the measurement of baseline HBV antibodies should be implemented when commencing immunoglobulin infusion. If the test results are negative, and there is an absence of hepatitis or risk factors, any future positive results in the context of ongoing immunoglobulin therapy should be considered false positives, HBV-DNA levels should be measured, and antiviral treatment should be given cautiously.

Since the introduction of adequate management and advanced testing technologies, no cases of IVIG-related prion disease or HIV transmission have been reported. Radomski et al. (112) found that two dedicated and one supplemental step [solvent/detergent (S/D) treatment and nanofiltration (20 nm) in combination with ion-exchange chromatography] could prevent pathogen transmission. Although the infection risk is much lower in IVIG than in other blood products, the possibility of infection can never be neglected.

#### Other Adverse Effects

Other adverse effects of immunoglobulin therapy include uveitis, passively acquired thyroid autoantibodies and reversible splenial lesion syndrome. Kocak et al. (113) reported a case in which a 44-year-old female developed bilateral uveitis following the administration of IVIG for 2 days. The patient was treated with topical corticosteroids and had achieved complete resolution at 1-month follow-up. In 2017, Uchida et al. (114) reported two cases in which thyroid autoantibodies passively acquired following IVIG administration. Finally, Uygur et al. (115) described a case of reversible splenial lesion syndrome caused by IVIG therapy.

## Preventive Measures

### Risk Assessment and Adequate Monitoring

Immunoglobulins cause various adverse effects; some of these effects are severe and fatal. Hence, a detailed medical history should be obtained in every patient being considered for immunoglobulin treatment. This information should include age, the infusion course of immunoglobulin, concomitant diseases (diabetes, hypertension, coronary heart disease, stroke, thrombotic events, hemadostenosis, etc.), and co-medications (e.g., contraceptive drugs and diuretics). Laboratory tests are also needed, such as a blood group test, routine blood tests, and tests of liver and kidney function. A variety of risk factors must be considered when evaluating possible adverse effects, and special precautions should be considered in patients with a history of allergies or thrombotic events. In these patients who have a heightened risk of developing adverse effects, special monitoring should be employed within the first 24 h following immunoglobulin administration in a hospital. The proposed factors that predispose a patient to immunoglobulin-induced adverse effects are shown in Table 2.

**Table 2:** Predisposing factors for immunoglobulin-induced adverse effects.

Adverse effect	Predisposing factors
Flu-like symptoms	High dose, rapid infusion rate, accompanying infection, previous adverse effects
Dermatological adverse effects	High dose, rapid infusion rate, accompanying infection, male patients with chronic inflammatory demyelinating polyneuropathy
Arrhythmia and hypotension	History of heart disease
Transfusion-related acute lung injury	Rapid infusion rate
Thrombotic events	High dose, rapid infusion rate, advanced age, being bedridden, diabetes mellitus, hypertension, dyslipidemia, prior/current thrombosis, preexisting atherosclerotic disease, elevated serum viscosity, oral contraceptive use, hereditary hypercoagulable state, idiopathic thrombocytopenic purpura
Aseptic meningitis	High dose
Renal impairment	Rapid infusion rate, advanced age, renal insufficiency, nephrotic syndrome, diabetes mellitus, dehydration, sepsis paraproteinemia, nephrotoxic drugs, hemolysis, sucrose-containing preparations
Hemolysis	High dose, rapid infusion rate, non-O blood group, underlying inflammatory state

### Slowing Down the Infusion Rate

The adverse effects described here are closely related to the rate of immunoglobulin infusion. Hence, slowing down the rate of infusion can greatly reduce the rate of adverse reactions, especially flu-like symptoms, hemolysis, thrombosis, and renal impairment (22, 63, 116). Strictly controlling the infusion rate during the first administration is recommended. During the first infusion, an initial slower rate should be implemented for the first 30 min, and then the rate may be increased if no adverse effects occur (we recommend using the infusion rate according to the instructions provided by different brands). Slowing the rate should be considered if any adverse effects occur. The infusion should be discontinued if slowing the rate does not alleviate these adverse reactions.

### Premedication and Prehydration

Premedication with antihistamines, corticosteroids, or NSAIDs can markedly reduce the severity and incidence of IVIG-induced adverse effects. In 1998, Roberton et al. (117) assessed the effect of premedication with methylprednisolone in a large crossover study that included 10 patients who had previously experienced frequent adverse reactions. Methylprednisolone was administered at a dose of 1 mg/kg 20 min prior to IVIG infusion, and the pretreated patients exhibited a marked decrease in the severity of IVIG-induced immediate adverse events (*P* < 0.01), with only one patient discontinuing IVIG infusion, whereas IVIG was interrupted in 8 of the 10 patients who were not pretreated with methylprednisolone. Souayah et al. (118) examined the safety profile of home infusion of IVIG in patients with neuroimmunologic disorders. In all, 276 patients were premedicated with antihistamines, corticosteroids, or NSAIDs, and the incidence of IVIG-induced adverse effects was significantly lower in the premedicated group than in the non-premedicated group (18.4 VS 27.1%, *P* = 0.04). However, the results of a separate study indicated that the rate of immunoglobulin-induced immediate adverse events was not altered by premedication (29). With regards to thrombotic events, Huang et al. (119) implemented a protocol in which treatment with antiplatelets and anticoagulation before IVIG infusion eliminated IVIG-related thrombotic events. However, because their sample size was small, further studies are needed to determine the safety and efficacy of this protocol.

Prehydration with normal saline is also used to prevent immunoglobulin-induced adverse effects. Many studies have proposed that prehydration can be helpful for headache, thrombolysis, renal impairment, and hemolysis (63, 120, 121); however, the implementation protocol, the dose of saline (250 or 500 ml, etc.), and the duration of saline infusion remain unclear. All of these studies utilized a protocol that combined hydration and other measures; therefore, the efficacy of prehydration should be further evaluated in a well-designed study.

### Switching From IVIG to SCIG or Other Immunoglobulin Preparations

Switching from IVIG to SCIG seems to be an effective strategy that attenuates immunoglobulin-induced adverse effects, especially for patients who have previously experienced severe adverse effects or are at high risk of developing adverse effects. An increasing number of well-designed studies show that SCIG can be used as a treatment for immunodeficiency diseases, multifocal motor neuropathy, chronic inflammatory demyelinating polyneuropathy, and myasthenia gravis (6, 122–136) (Table 3). The results of two randomized, crossover studies indicate that the rate of systematic adverse effects was lower following SCIG than following IVIG, and no severe adverse effects were reported in patients treated with SCIG (123, 135). However, the sample sizes of these two studies were small (30 and 20). Racosta et al. (137) performed a meta-analysis of reports that explored the efficacy and safety of SCIG VS IVIG and identified a total of 8 studies comprising 138 patients with inflammatory demyelinating polyneuropathies. Their results showed that the relative risk of moderate and/or systemic adverse effects was 28% lower in the SCIG group [95% confidence interval, 0.11–0.76]. Due to the small sample size of these studies, the efficiency of SCIG should be further explored, and more randomized controlled trials with larger samples of patients with various diseases are needed.

**Table 3:** Subcutaneous immunoglobulin (SCIG) treatment in a variety of diseases.

Reference	Sample	Diagnosis	Study design	SCIG dose	Rate of side effects	Severity
Misbah et al. (130)	8	Multifocal motor neuropathy	Prospective, open-label, multicenter study	271.8 ± 139.13 mg/kg (range, 100–488 mg/kg)	50% had 18 AEs 0.098 per infusion	Mild to moderate No discontinuations

Kanegane et al. (133)	25	Primary immunodeficiency	Prospective, multicenter, open-label, single-arm study	Not mentioned	96.0% had 269 AEs 0.461 per infusion	Mild to moderate No discontinuations

Empson et al. (131)	35	Primary immunodeficiency	Phase III, single-arm, open-label, multicenter study	Median 6.70 g/week (range, 3–13.5 g/week)	40% of patients 0.059 per infusion	Mild to moderate No discontinuations

Hoffmann et al. (128)	82	Primary and secondary antibody deficiencies	Prospective, observational, multicenter study	91 ± 31 mg/kg/week	2/82 patients had 2AEs	Local tissue reactions 1 patient discontinued

Thépot et al. (129)	65	Primary hypogammaglobulinemia	Monocentric, longitudinal trial	108 mg/kg (range, 62–174 mg/kg)	3/65 patients	Mild to moderate 3 patients switched to intravenous immunoglobulin

Berger et al. (126)	51	Primary immunodeficiency	Not mentioned	100–200 mg/kg/week	86.3% of patients 0.15 per infusion	1 severe adverse effect 2 patients withdrew

Spadaro et al. (134)	14	Hypogammaglobulinemia	Not mentioned	100 ± 4.4 mg/kg/week	Not mentioned	Not mentioned

Harbo et al. (127)	6	Multifocal motor neuropathy	Prospective, observational study	13–51 g per week	6/6 patients had local tissue reactions	Local tissue reactions 1 patient withdrew

Ochs et al. (124)	65	Primary immunodeficiency	Prospective, open-label, multicenter study	158 mg/kg (range, 155–165 mg/kg)	60/65 patients 0.52 per infusion	Mild to moderate 14 patients withdrew

Markvardsen et al. (132)	20	Chronic inflammatory demyelinating polyneuropathy	Randomized, single-blind, crossover study	0.4 g/kg	Not mentioned	Not mentioned

van Schaik et al. (136)	172	Chronic inflammatory demyelinating polyneuropathy	Randomized, multicenter, double-blind, placebo-controlled, parallel-group, phase III study	0.2 or 0.4 g/kg	30% in the low-dose group 34% in the high-dose group	Six (3%) patients had 11 serious adverse events

Beecher et al. (6)	23	Myasthenia gravis	Prospective, open-label, phase 3 trial	2 g/kg	Headache and injection site reactions were common	Mild to moderate No discontinuations

Markvardsen et al. (135)	30	Chronic inflammatory demyelinating polyneuropathy	Randomized, double-blind, placebo-controlled trial	4.8–48 g/week	6/15 patients	Mild to moderate No discontinuations

Gardulf et al. (122)	165	Primary immunodeficiency	Not mentioned	Not mentioned	17% of patients 0.03 per infusion	Mild to moderate No discontinuations

Eftimov et al. (125)	10	Multifocal motor neuropathy	Prospective, open-label, noncontrolled study	0.46 g/kg/month (range, 0.27–0.62)	9 patients developed local adverse events	Mild to moderate No discontinuations

Chapel et al. (123)	30	Primary immunodeficiency	Randomized, multicenter, open-label, crossover trial	Not mentioned	0.1 per infusion	Mild to moderate No discontinuations

It remains unclear how the dose should be adjusted when switching from IVIG to SCIG. Berger and colleagues found that using 137 and 153% of the IVIG dose when switching to SCIG produced the same effect in patients being treated for primary immunodeficiencies (138). Another study concluded that sustained serum IgG levels can be achieved after switching to SCIG despite the use of a reduced immunoglobulin dose in patients with primary hypogammaglobulinemia (129). Individualizing the dosage based on the disease state and the clinical response is preferable to using mean pharmacokinetic parameters when switching from IVIG to SCIG. In addition, more studies focused on pharmacokinetics in immune-mediated diseases are needed.

Detailed information regarding the immunoglobulin preparation should always be recorded, including the manufacturer, batch, and drug instructions. It is clear that the content, composition, and characteristics of each immunoglobulin preparation can adversely affect patients in different manners. Hence, if patients frequently develop adverse effects following administration with an IVIG preparation, switching to another immunoglobulin preparation may lead to fewer adverse effects (62).

### Other Measures

Many patients develop immunoglobulin-associated adverse effects, and the majority of these effects are mild to moderate and resolve with appropriate treatment. The observed effects include immediate adverse effects, aseptic meningitis, hemolysis, neutropenia, and electrolyte disturbances. Some of these severe adverse effects should be treated according to the principle underlying the corresponding diseases, such as an effective antithrombotic therapy in patients with thrombosis or short-term renal replacement therapy in patients with severely damaged renal function.

## Future Prospects

Immunoglobulin preparations have been widely used in a variety of diseases, but controlled studies have not been performed for many diseases, such as myasthenia gravis, some forms of lupus erythematosus, septic syndrome, and polymyositis. The improper usage of immunoglobulin increases the risk of adverse effects to some extent; therefore, further studies are needed to demonstrate the proper indications for the use of immunoglobulin.

The majority of adverse effects are associated with high doses of immunoglobulin; thus, determining individualized dosages to guarantee the efficacy of therapy and minimize adverse effects is an urgent focus. Ameratunga (139) suggested that the initial IVIG dose should be based on adjusted body weight in obese patients with primary immunodeficiency disorders. As this study was a single observation and focused on only obese patients, additional special populations, such as the elderly, should be studied to determine the parameters of individualized dosing for immunoglobulin therapy.

Many measures have been used to prevent or minimize immunoglobulin-related adverse effects, as summarized above. Given that these studies were case controlled or small sample size studies, the efficacy of these measures should be verified by randomized controlled studies or head-to-head studies with larger sample sizes. Simultaneously, the use of only one preventive measure may not prevent adverse effects, and a series of measures serving as a standard protocol may be effective for preventing serious adverse effects, such as combining prehydration with anti-thrombosis to minimize thrombotic events.

Currently, immunoglobulin may be given intravenously or subcutaneously to treat a variety of disorders. However, IVIG and SCIG preparations also result in adverse effects. It is possible that other routes of immunoglobulin administration may reduce the rate of adverse effects. In 1982, Barnes et al. (140) found that oral administration of immunoglobulin could be used for the prevention and treatment of rotavirus diarrhea in low-birth weight babies. Later, several meta-analysis studies concluded that oral administration of immunoglobulin could not prevent rotavirus diarrhea and necrotizing enterocolitis (141, 142). Although these studies have several limitations (small sample size and few well-designed studies), oral administration may be a promising preparation to attenuate the occurrence of adverse effects.

Many factors affect the rate of immunoglobulin-related adverse effects. Several characteristics of newer generation immunoglobulin products should be improved, including specific functions, purity, and biological safety. Thus, advanced separation and purification technologies should be developed.

## Author Contributions

YG and XT conceived the article and wrote the manuscript. XW and ZX reviewed and edited the manuscript. All authors read and approved the manuscript.

## Conflict of Interest Statement

The authors declare that the research was conducted in the absence of any commercial or financial relationships that could be construed as a potential conflict of interest.
